# Response evaluation after neoadjuvant treatment for rectal cancer using modern MR imaging: a pictorial review

**DOI:** 10.1186/s13244-019-0706-x

**Published:** 2019-02-13

**Authors:** Doenja M. J. Lambregts, Thierry N. Boellaard, Regina G. H. Beets-Tan

**Affiliations:** 1 Department of Radiology, Antoni van Leeuwenhoek - Netherlands Cancer Institute, Amsterdam, The Netherlands; 2 GROW School for Oncology and Developmental Biology - Maastricht University, Maastricht, The Netherlands

**Keywords:** Rectal neoplasms, Magnetic resonance imaging, Diffusion magnetic resonance imaging, Chemoradiotherapy

## Abstract

In recent years, neoadjuvant chemoradiotherapy (CRT) has become the standard of care for patients with locally advanced rectal cancer. Until recently, patients routinely proceeded to surgical resection after CRT, regardless of the response. Nowadays, treatment is tailored depending on the response to chemoradiotherapy. In patients that respond very well to CRT, organ-preserving treatments such as watch-and-wait are increasingly considered as an alternative to surgery. To facilitate such personalized treatment planning, there is now an increased demand for more detailed radiological response evaluation after chemoradiation. MRI is one of the main tools used to assess response, but has difficulties in assessing response within areas of post-radiation fibrosis. Hence, MR sequences such as diffusion-weighted imaging are increasingly adopted in clinical MR protocols to improve the differentiation between tumor and fibrosis. In this pictorial review, we discuss the strengths and weaknesses of modern MR imaging, including functional imaging sequences such as diffusion-weighted MRI, for response evaluation after chemoradiation treatment and provide the main pearls and pitfalls for image interpretation.

## Keypoints

- Response evaluation with MR imaging can serve as a surgical roadmap and help identify (near-)complete responders for organ-preserving treatments
- Diffusion-weighted MRI improves the performance of MRI to discriminate between tumor and fibrosis, but certain pitfalls need to be taken into account
- Knowledge on specific patterns of morphology and diffusion signal can be helpful to improve response evaluation with MRI

## Introduction and clinical background

Neoadjuvant therapy has become the standard of care for patients with locally advanced rectal cancer. Neoadjuvant treatment can consist of a short course of radiotherapy (5 × 5 Gy) +/− a prolonged waiting interval or a long course of combined chemoradiotherapy (CRT) [1]. The main aim of CRT is to downsize and downstage the tumor to increase the chance of a complete resection and consequently reduce the local recurrence risk. Moreover, organ-preserving treatment strategies (local excision or ‘watch-and-wait’) have recently been introduced as a potential option for patients that show a (near-)complete response to CRT.

Magnetic resonance imaging (MRI) is since many years considered pivotal for the staging and treatment planning of primary rectal tumors. Nowadays, MRI is also increasingly incorporated in clinical routine to assess response and restage tumors after CRT. While in the past the surgical strategy was mainly determined based on the findings of primary staging, the findings of restaging MRI are now increasingly used to guide further treatment. Current guidelines therefore recommend to routinely perform MRI for restaging of rectal cancer after CRT [2].

Restaging after CRT can impact treatment planning in two ways:

First, the findings on post-CRT MRI can serve as a ‘roadmap’ to optimize the surgical strategy. In distal tumors that primarily invaded the anal sphincter, downsizing may cause the tumor to retract from the sphincter, thereby allowing sphincter-preserving surgery after CRT. Similarly, retraction from initially invaded organs in T4 tumors may allow conversion from extended resection into standard total mesorectal excision (TME).

Second, with the current paradigm shift toward organ-preserving treatment strategies, MRI—together with digital rectal examination and endoscopy—can have a role in selecting the right candidates. About 15–25% of patients undergo a complete response after CRT [3]. When these complete responding patients are accurately selected, surgery with its associated morbidity and even mortality can be omitted with good results in terms of overall and disease-free survival as well as with improved quality of life in recently published studies [4–6]. When patients are included in a watch-and-wait program, imaging can also play a role in patient monitoring. Although the intensity of follow-up and modalities used differ between published studies, patients are typically regularly monitored with the aim to detect tumor recurrences as early as possible so that salvage surgery can still be performed. These patients have shown comparable outcomes compared to patients operated immediately after CRT [5]. MRI is routinely included in the follow-up program in several reports and can be particularly beneficial for the detection of mesorectal (e.g., nodal) sites of recurrence [5, 6].

These developments increase the demand for an accurate radiologic evaluation of response. With this pictorial review, we discuss the strengths and weaknesses of modern MRI imaging, including functional imaging sequences such as diffusion-weighted MRI, for response evaluation after chemoradiation treatment and provide the main pearls and pitfalls for image interpretation.

## Protocol and patient preparation

**Fig. 1:**
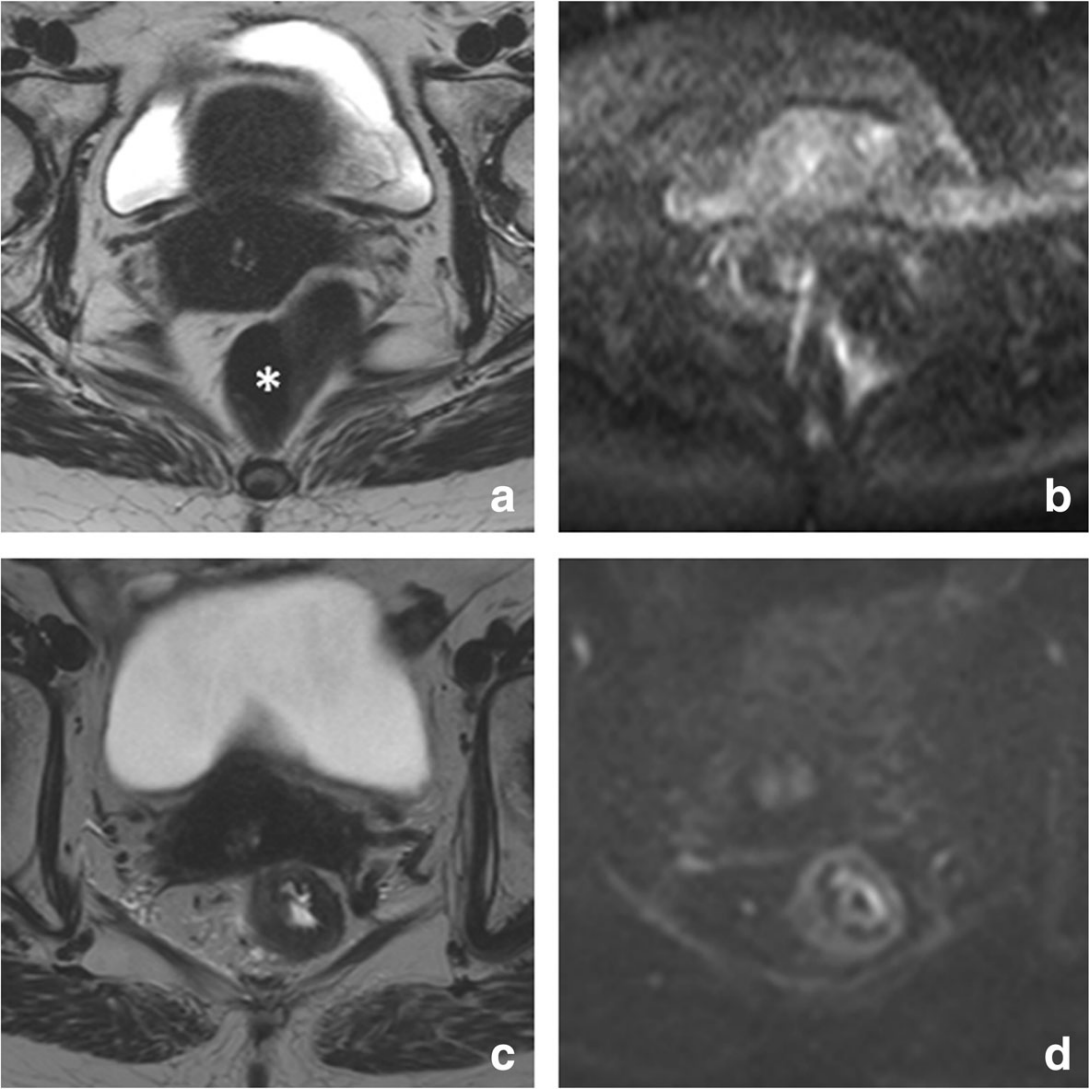
Effect of preparatory micro-enema. MR images of the same patient performed without (**a** T2-weighted; **b** b1000 diffusion-weighted) and with (**c** T2-weighted; **d** b1000 diffusion-weighted) a preparatory micro-enema. The patient is a clinical complete responder, followed with MRI as part of a watch and wait-program. Note the severe distortions on DWI (**b**) caused by gas in the rectal lumen (* in **a**). On the MRI performed after a preparatory micro-enema (self-administered by the patient 15 min before the MRI), there is no gas present in the rectum and the rectal wall is well discernible, both on T2-weighted and diffusion-weighted MRI

The recommended MR imaging protocol for restaging is similar to that for primary staging. Two-dimensional T2-weighted sequences in three planes are the mainstay of the protocol. In addition, recent consensus guidelines from the European Society of Gastrointestinal and Abdominal Radiology (ESGAR) recommend to routinely include a diffusion-weighted imaging (DWI) sequence in the restaging protocol [2]. Transverse sequences should be angulated perpendicular to the (former) tumor axis and the coronal series parallel. For distal tumors, a coronal sequence angulated to the anal canal is mandatory to assess the presence and extension of anal sphincter invasion. Spasmolytics can be used to reduce bowel movement artifacts. Although not mandatory for all cases, spasmolytics are particularly useful in upper rectal tumors where bowel movements can significantly reduce image quality. Further patient preparation is not required, although the ESGAR consensus panel acknowledged that preparatory steps to reduce the amount of gas in the rectal lumen (such as a preparatory micro-enema or small volume of rectal filling (up to 60 ml)) may be helpful to avoid artifacts on DWI-sequences that are prone to gas-induced susceptibility effects. Evidence on the benefit of these approaches is however scarce and needs to be further established [7]. An example of a patient imaged with and without a preparatory micro-enema is provided in Fig. 1. Filling the rectal with higher volumes is not recommended because this causes rectal wall distension and compression of perirectal tissues which can hamper correct interpretation of the relation of the tumor with surrounding structures [8]. There are no clear guidelines regarding the optimal timing after CRT to perform the restaging MRI. Recent literature typically reports intervals of ± 8 weeks after completion of CRT, although prolonged intervals may be useful to enhance response rates [9, 10]. Whether and with what frequency MRI should be performed in patients monitored as part of an organ-preservation program is not yet clearly established. In studies that included MRI, the frequency varied from routinely every 3 to 6 months [4] to yearly [5]. Future studies should focus on further determining the optimal modalities, timing, and frequency of follow-up, which is an important issue given the patient burden, but also economic burden and manpower required associated with repeated imaging.

## Morphologic evaluation of tumor response

As a result of successful response to treatment, rectal tumors typically decrease in size while undergoing a fibrotic transformation. Untreated (non-mucinous) tumors show intermediate signal intensity on T2-weighted MRI that is lower than that of fat tissue, but higher than that of the normal muscular layer of the bowel wall. When tumor tissue becomes fibrotic, the signal drops considerably and the tumor bed becomes markedly hypointense. A small minority of tumors develop a mucinous response as a result of CRT, leading to an increase in signal after CRT [11]. At histopathology, these ‘mucin’ lakes typically contain no or rare isolated tumor cells [12]. A mucinous transformation in primarily non-mucinous tumors should therefore be considered a good prognostic sign. This should not be confused with primarily mucinous tumors that have an overall poorer prognosis and tend to show a poor response to CRT (see separate section on ‘Mucinous and signet-ring subtype’ below). Reductions in tumor volume in the range of 60–80% have been reported in literature to correlate with a good response to treatment [13]. Although in oncology Response Evaluation Criteria in Solid Tumors (RECIST) is often used as a unidimensional size measurement system to assess response, RECIST is not commonly used in rectal cancer, because it can be difficult to reproducibly measure irregularly shaped rectal tumors in one plane. 3D whole volume tumor measurements have been reported to be better reproducible and more accurate to assess response [13], but these measurements can be very time consuming. As a practical alternative, the recent ESGAR guidelines therefore suggest to measure the tumor length before and after CRT, as reported measurement reproducibility for this metric is good and it at least offers some estimation of the change in tumor size/volume as a result of treatment [2].

### MR tumor regression grade

Although not (yet) routinely adopted for rectal cancer restaging worldwide, the degree of fibrotic transformation as a measure of response can also be classified using the MRI tumor regression grade (mrTRG), which is an imaging adaptation of similar TRG systems commonly used in histopathology [14]. The mrTRG grades the degree of fibrotic response on T2-weighted MRI using a 5-point scale:

- TRG1 = thin low signal fibrosis with no evidence of intermediate signal intensity
- TRG2 = dense low signal fibrosis with no evidence of intermediate signal intensity
- TRG3 = predominant low signal fibrosis with scattered or focal intermediate signal intensity
- TRG4 = predominant intermediate signal intensity with minimal fibrosis
- TRG5 = intermediate signal intensity with no evidence of fibrosis

Interobserver agreement for assessment of mrTRG has been reported to be good [15], but agreement between the mrTRG and pathologic TRG rather low and the performance of mrTRG to identify complete responders has been shown to be limited with reported sensitivity of 74% and specificity of 63% [16]. The mrTRG has however been shown (mainly by reports from the UK) to be beneficial as a biomarker to distinguish between good and poor response groups and to predict survival outcomes [17–20]. We are currently awaiting the results of the first randomized trial from the UK to stratify treatment management based on the mrTRG as a biomarker to select good and poor responders [21]. One group introduced a modified mrTRG system for mucinous tumors which also showed a correlation with pathologic treatment response [22]. Another group recently introduced a modified mrTRG system incorporating DWI findings and suggested a benefit to predict prognosis [23]. Results of these single reports remain to be validated.

### Morphologic patterns of response

**Fig. 2:**
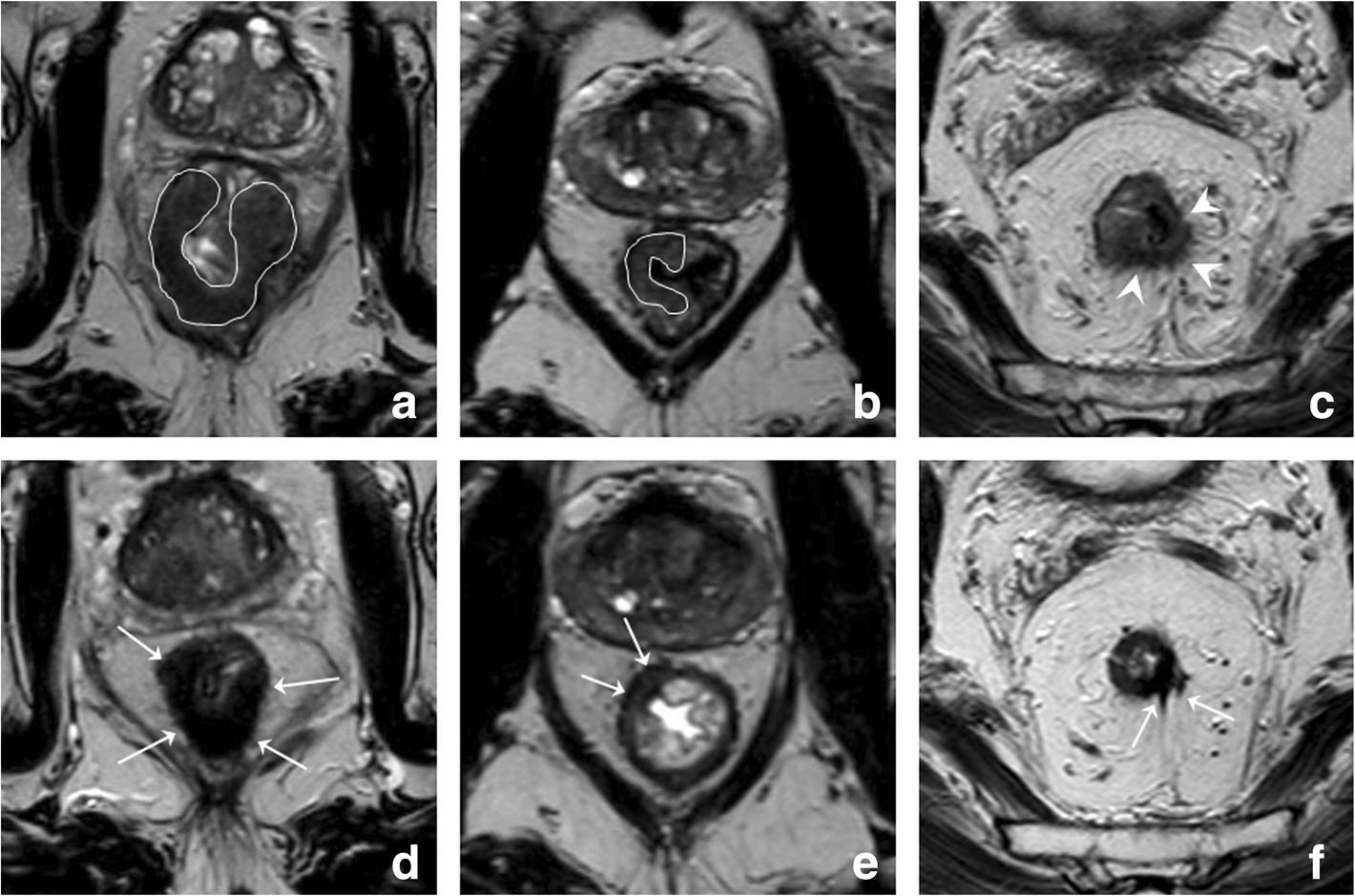
Patterns of fibrosis. Examples of the pre-CRT (**a**–**c**) and corresponding post-CRT (**d**–**f**) T2-weighted images of three male patients with low to mid-rectal tumors. The first patient has a well-defined, almost circular tumor mass (**a**). After CRT (**d**), the tumor has undergone fibrotic transformation that follows the shape of the primary tumor, resulting in a full thickness (semi) circular fibrotic wall thickening. The second patient has a relatively small, semicircular tumor (**b**). After CRT, only a small focal area of fibrosis remains visible that is confined to the rectal wall (**e**). The third patient has an irregularly shaped, spiculated tumor (**c**). After CRT, the fibrosis also has an irregular aspect with persistent spiculations (**f**)

The degree of response to chemoradiotherapy in solid tumors can vary from a poor response with an obvious solid residual tumor mass after CRT at one end of the spectrum, to a complete normalization of the rectal wall without any visible mass or fibrosis at the other end. A recent study has shown that in such cases, radiologists can make a confident diagnosis of either residual tumor or a complete response, respectively [24] These clear-cut cases, however, comprise the extreme ends of the spectrum and only occur in a reported ± 5% (normalized wall) and ± 20% (bulky residual mass) of patients [24]. In the remaining majority, varying degrees of volume reduction and fibrotic transformation are observed. The pattern of fibrosis typically follows that of the primary tumor: irregular/spiculated tumors tend to also show irregular fibrosis after CRT, while more sharply demarcated (semi) circular or polypoid tumors tend to show more sharply demarcated focal fibrosis. Examples of these various patterns of fibrosis are illustrated in Fig. 2 [24, 25]. Patterns where only a small, well-defined fibrotic remnant remains visible within the rectal wall after CRT are more often associated with a complete response [25], while very irregular fibrosis patterns more frequently contain residual tumor [24]. Although these patterns can be helpful, the differentiation of vital tumor within the fibrosis on an individual patient level remains a challenge. Studies have shown low sensitivities ranging between 11 and 71% for routine T2-weighted MRI to identify complete responders [26–30], with a pooled sensitivity of only 19% in a recent meta-analysis [31]. These results indicate that with standard MRI, many complete responders are overstaged, reflecting the tendency of radiologists to err on the safe side when they see fibrosis after CRT. This drawback greatly limits the usefulness of morphological MRI to help select patients for organ-preserving treatments.

### Morphologic MRI as a surgical roadmap post-CRT

**Fig. 3:**
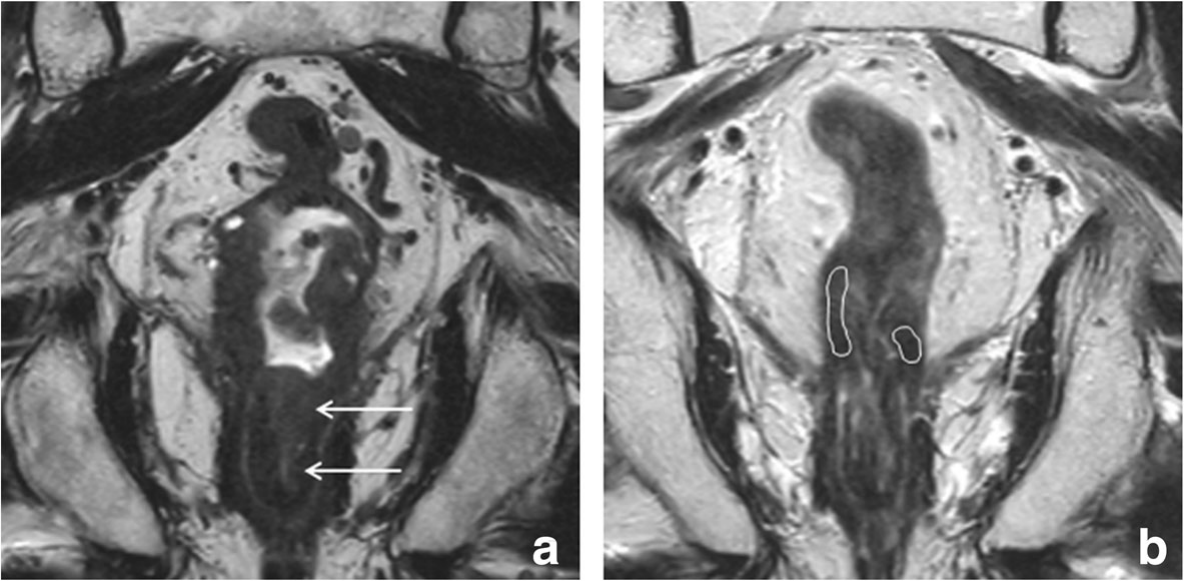
Assessment of sphincter involvement. Coronal T2-weighted MR images of a male patient with a tumor in the distal rectum before (**a**) and after CRT (**b**). Before treatment the tumor extends into the proximal one third of the anal canal where it invades the internal anal sphincter on the left lateral side (arrow in **a**). After CRT, the tumor has decreased considerably in size and retracted from the anal canal. A fibrotic remnant (circles in **b**) remains visible above the level of the anorectal junction. The anal sphincter is no longer involved

As described above, MRI has known difficulties in differentiating between fibrosis still containing vital tumor cells and mere fibrosis. Nevertheless, MRI can be helpful to surgeons to determine their operative strategy after CRT. In patients with suspected tumor involvement of the anal sphincter before treatment, MRI can help in determining if the sphincter remains invaded after CRT (see Fig. 3). This information can help surgeons to determine whether or not sphincter-preserving surgery will be feasible. In a (preliminary) report, it was shown that MRI can predict feasibility of successful sphincter preservation with an overall diagnostic performance (area under the ROC-curve) of 0.84–0.87 [32].

**Fig. 4:**
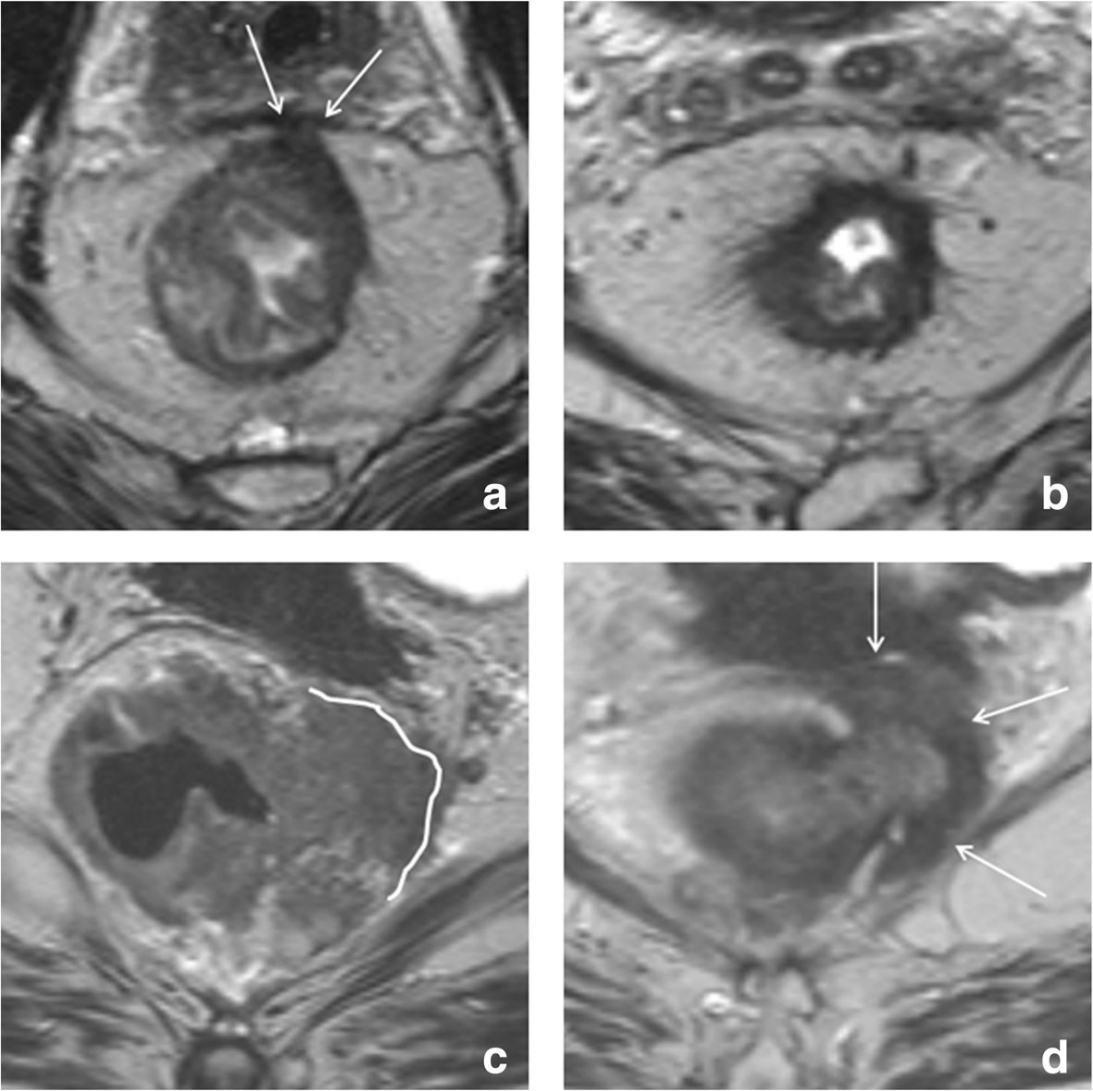
Patterns to predict mesorectal fascia involvement after CRT. Example of two morphologic patterns that can aid in predicting MRF involvement after CRT. The male patient in the upper row has a mid-rectal tumor that invades the MRF anteriorly (arrows in **a**). After CRT (**b**), the tumor has retracted from the MRF, only some slight fibrotic stranding towards the MRF remains. Subtle fibrotic stranding (or a cleared fat plane) are signs indicating a high chance of a tumor-free MRF, which was indeed confirmed at histopathology after surgery in this patient. The female patient in the bottom row has a mid-rectal tumor with extensive MRF invasion at the left lateral side (white line in **c**). After CRT (**d**), the tumor has decreased in volume, but there is still an isointense mass surrounded by hypointense fibrosis that involves the MRF from 1 to 4 o’clock (arrows in **d**). This type of diffuse MRF infiltration reliably predicts persistent MRF involvement, which was indeed confirmed at histopathology after surgery in this patient

Similarly, MRI can aid in assessing tumor retraction from previously invaded adjacent pelvic organs in T4 tumors [33]. Results for MRI to predict clearance of the mesorectal fascia after CRT vary in literature. Although MRI is known to be highly accurate in predicting involvement of the MRF at primary staging [34, 35], assessment after CRT is more difficult owing to the presence of fibrosis. In the meta-analysis by van der Paardt et al., MRI had a moderate performance for predicting a tumor-free MRF after CRT with a sensitivity of 76% and specificity of 86% [31]. Vliegen et al. described certain morphologic patterns that can help the radiologist (Fig. 4). When a clear fat plane, or a fat plane containing only minimal fibrotic strands re-appears between the tumor and MRF after CRT (Fig. 4a, b), there is a negligible risk of persistent MRF involvement at histopathology. Conversely, when there is still diffuse T2 isointense and/or hyperintense tissue infiltration of the MRF after CRT (Fig. 4c, d), the risk for tumor invasion at histopathology is 90%. The most difficult cases are those with diffuse fibrotic infiltration of the MRF. In these cases, the risk for MRF positivity at histopathology is around 50% [36].

### Mucinous and signet-ring subtype

**Fig. 5:**
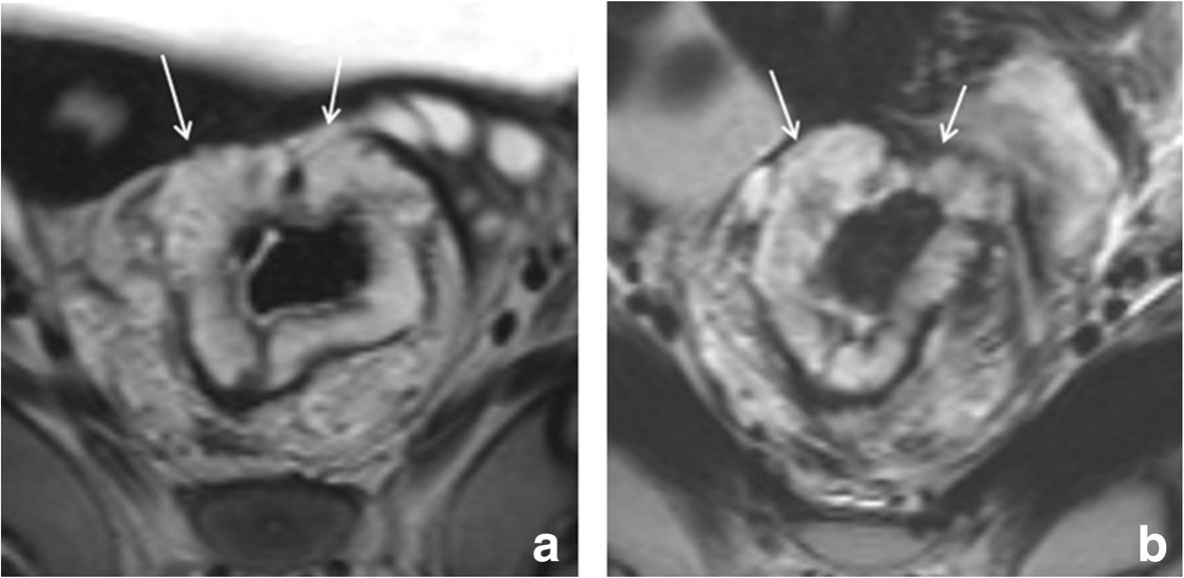
Response assessment in mucinous type adenocarcinoma. Example of the pre- (**a**) and post-chemoradiotherapy (**b**) T2-weighted images of a young female patient with a mucinous type rectal adenocarcinoma. The tumor presents as a circular mass with distinctly high T2 signal intensity. Note the extensive MRF involvement anteriorly (arrows). The tumor has a close relation to the cervix but does not invade it macroscopically. After CRT, the volume and morphology of the mass is largely unchanged on MRI. On MRI, it is difficult to discriminate between sterilized mucin and mucin still containing viable tumor cells. Histopathology showed persistent ypT3N2 tumor

**Fig. 6:**
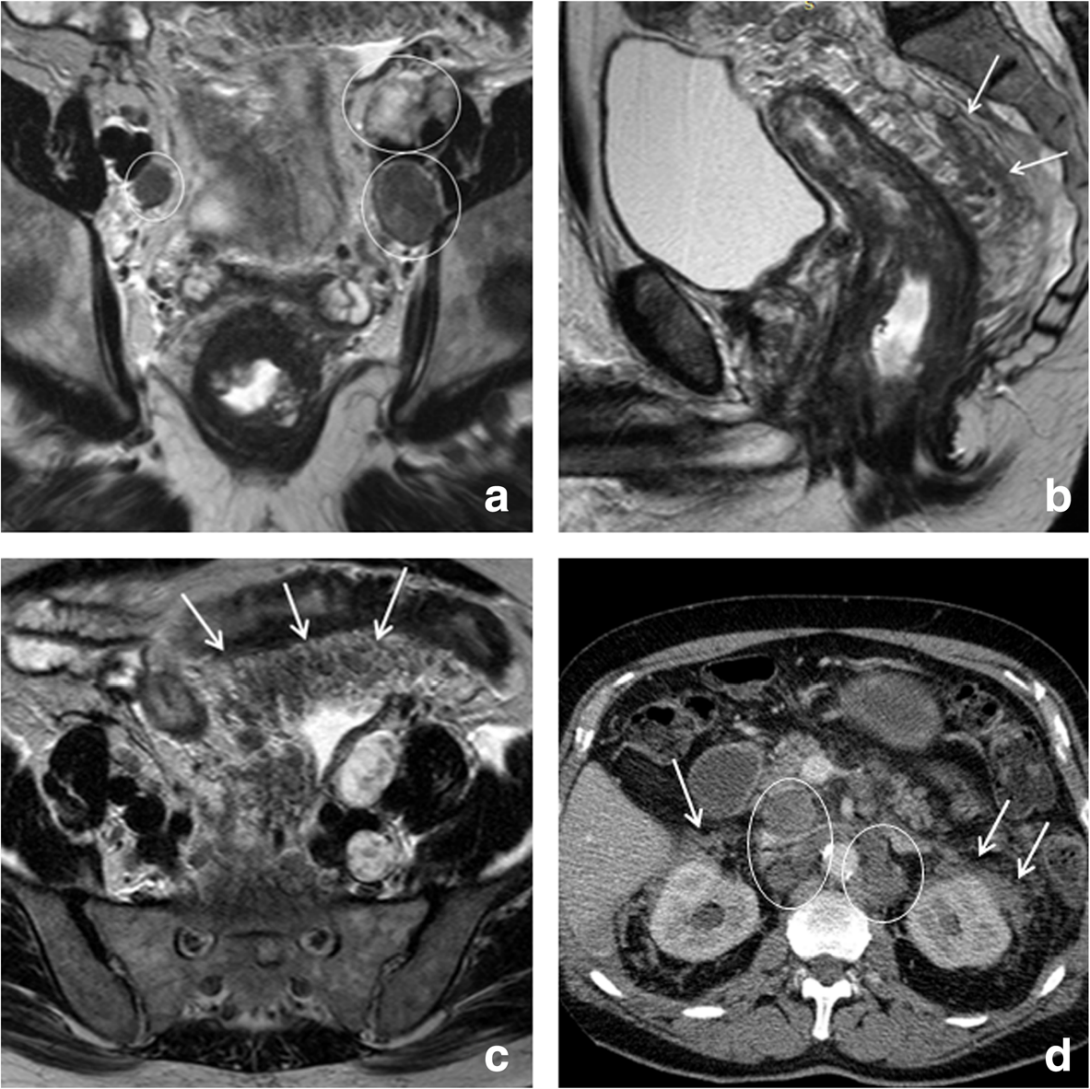
Signet-ring tumor. Transverse (**a**), sagittal (**b**) and coronal (**c**) T2-weighted MR images, and CT image (**d**) of a male patient with a signet-ring carcinoma of the rectum. Note the typical diffuse hypointense thickening of the rectal wall with extensive tumor infiltration in the mesorectal fat (arrows in **b**), that extends all the way up cranially into the mesosigmoidal fat (arrows in **c**). There are several large metastatic lymph nodes both in the pelvis (circles in **a**) as well as para-aortic in the upper abdomen (circles in **d**). At the time of diagnosis, the patient presented with extensive peritoneal metastases (arrows in **d**). Palliative chemotherapy was started, shortly after which the patient deceased

Mucinous adenocarcinoma is an important subtype of rectal cancer which comprises about 5–20% of all rectal cancers [37]. These tumors are characterized by extracellular mucin secretion and can easily be distinguished on MRI because they show distinctly high T2 signal intensity. Compared to non-mucinous (solid) adenocarcinomas, mucinous tumors typically show a relatively poor response to CRT [38]. Assessing the response of mucinous tumors on MRI is furthermore difficult, because they generally show no or only little shrinkage after CRT and because the high signal intensity remains, regardless of the histopathologic degree of viable residual tumor cells within the mucin (Fig. 5). As a result, the morphologic response criteria described above are typically poorly applicable in mucinous tumors. Mucinous tumors have a relatively high risk for metastases (34% versus 28% for common adenocarcinomas) [39]. A second, more rare histologic subtype (accounting for ± 1% of all rectal carcinomas) is the signet-ring cell carcinoma. Signet-ring tumors are associated with poor overall survival and typically present with extensive lymphatic and peritoneal tumor spread. They have the highest overall risk for distant metastases (59%) [39]. Given their poor prognosis and survival, signet-ring tumors are often palliatively treated, with consequently only a limited role for response evaluation with imaging. An MR example of a signet-ring tumor is provided in Fig. 6.

## Diffusion-weighted imaging

Diffusion-weighted imaging (DWI) is a technique that analyses how water molecules can move (‘diffuse’) in a certain tissue. Diffusion-weighting is achieved by applying diffusion-sensitizing gradients to a T2-weighted sequence. The degree of diffusion-weighting applied is referred to as the ‘*b* value,’ commonly in the range of b800–1000 s/mm^2^ for visual assessment. In normo- or low cellular tissue, water can move freely causing a decay of the signal on high *b* value images. In highly cellular tissues, the diffusion capacity of water is restricted and the signal is retained. This makes DWI a very suitable technique to detect malignant tumors, as has been demonstrated by numerous reports in various cancer types, including rectal cancer [40, 41]. Diffusion-weighted images are typically assessed in conjunction with the corresponding apparent diffusion coefficient (ADC) map. An ADC map is a parametric map that reflects the degree of water diffusivity for each voxel within the image, with a high signal representing free diffusion and a low signal representing restricted diffusion. It can also be used to derive quantitative diffusion-measurements (see section on ‘Quantitative functional imaging’ below).

### DWI for response evaluation: pearls and pitfalls

The basic principal of DWI interpretation on restaging MRI is fairly simple: high signal on DWI within the bowel wall or fibrosis at the location of the tumor bed indicates residual tumor, while the absence of signal is suggestive of a complete response. With this approach, several authors have shown that addition of DWI to standard T2-weighted sequences can significantly improve the performance of MRI to differentiate between patients with a complete tumor response and those with residual tumor [26–30]. In a meta-analysis, pooled sensitivity to predict response was significantly higher for studies including a DWI sequence (84%), compared to studies using only standard MRI (50%) [31].

There are, however, several pitfalls that may hamper correct interpretation of diffusion images. Misinterpretation of T2 shine-through effects, misinterpretation of low intensity on ADC maps, and misinterpretation of high signal caused by susceptibility artifacts are among the most common pitfalls encountered [42].

#### T2 shine-through

**Fig. 7:**
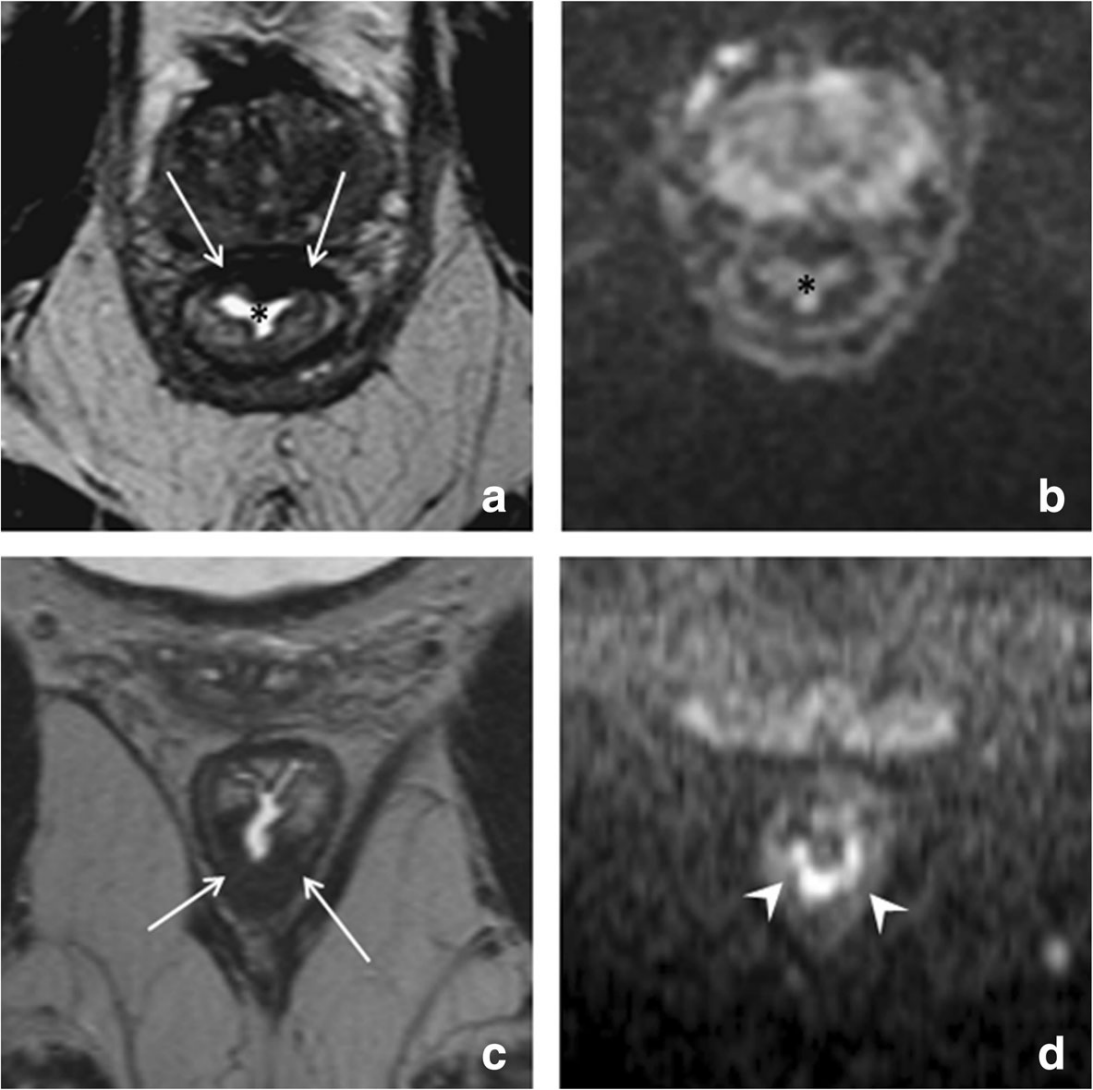
T2 shine-through. Post-CRT T2-weighted (**a**, **c**) and diffusion-weighted (**b**, **d**) images of two male patients who have been treated for low rectal tumors. The upper patient was a complete responder, while the patient in the bottom row still had a ypT2 tumor remnant at histopathology. A very similar fibrotic wall thickening (arrows in **a** and **c**) can be observed in both patients, reflecting the difficulties of T2-weighted MRI to assess response in case of fibrosis. In the upper patient (the complete responder), a star-shaped signal is observed in the rectal lumen (*), representing T2 shine-through of a small amount of fluid present within the rectal lumen. In the lower patient, there is a high signal with a more U-shaped configuration located at the inner margin of the fibrosis (arrowheads in **d**), representing the small ypT2 tumor remnant

T2 ‘shine-through’ refers to the presence of high T2 signal on DWI that is not caused by restricted diffusion. Diffusion-weighted images are inherently T2-weighted. As a result, tissues with a long T2-relaxation time (e.g., fluids) can appear bright, even in the absence of diffusion restriction. In rectal DWI, the main source of error is T2 shine-through occurring in small amounts of fluid in the rectal lumen, which can be mistaken for tumor in the adjacent rectal wall. To differentiate between luminal T2 shine-through and tumor, it is helpful to refer to the ADC map where luminal fluids will show a high signal because of a lack of true diffusion restriction. Moreover, the shape of the signal in the lumen has been described to be typically more star-shaped, as opposed to the more focal (mass-like) or U-shaped signal configuration in case of residual tumor (example shown in Fig. 7). T2 shine-through effects are also commonly observed in mucinous tumors that—owing to their high mucin content—exhibit very high signal on T2-weighted MRI that can be retained on DWI, making DWI a less suitable technique to assess mucinous tumors.

#### Low ADC signal in fibrosis (‘T2 dark-through’)

**Fig. 8:**
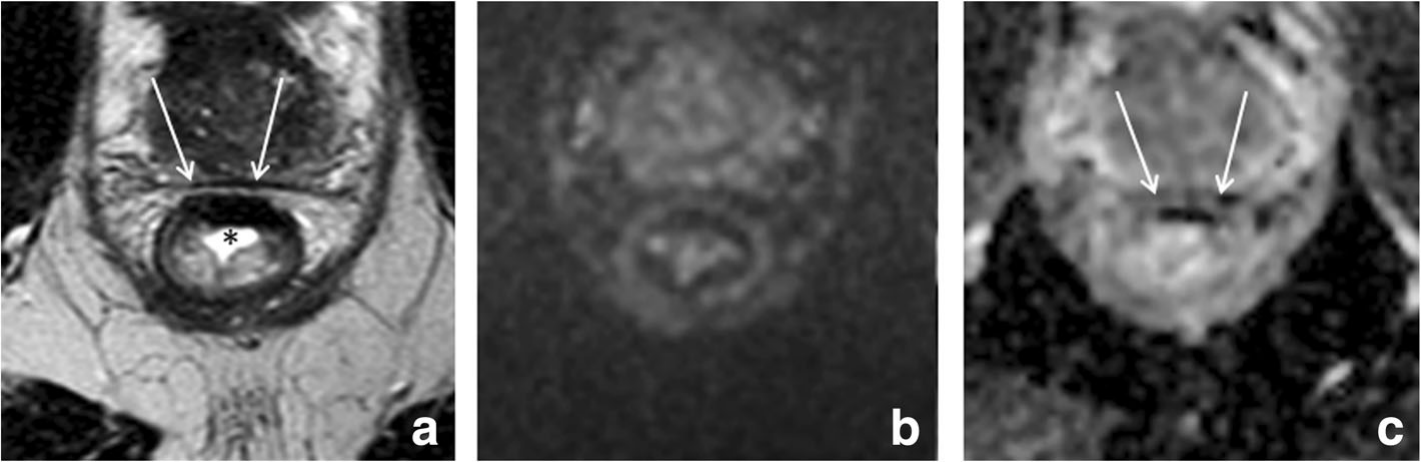
Pitfall of low ADC in fibrosis. Example of the post-CRT T2-weighted MRI (**a**), b1000 diffusion-weighted MRI (**b**), and ADC map (**c**) of a male patient treated for a low rectal tumor. The patient was a complete responder. A fibrotic wall thickening is present in the anterior rectal wall (arrows). On the ADC map (**c**), a markedly low signal is observed in the fibrosis, which is due to the high collagen content in fibrosis (that has a short T2 relaxation time) and should not be mistaken for restricted diffusion due to tumor. On DWI (**b**), the signal in the rectal wall is homogeneously low, indicating that there is no restricted diffusion. Note again the star-shaped shine-through of high T2 signal (*) caused by some fluid in the rectal lumen

Another potential pitfall is the markedly low signal that can be observed in areas of fibrosis on the ADC map. When solely looking at the ADC map, these low signal areas may be mistaken for residual tumor. The low signal in fibrosis is, however, mainly caused by the high collagen content of dense fibrosis, which has a short T2-relaxation time resulting in low signal on the T2-weighted MRI but also on DWI and the ADC map. This effect is sometimes referred to as ‘T2 dark-through’ or ‘T2 black out.’ An example is shown in Fig. 8. Low signal on the ADC map should therefore only be considered suspicious for tumor in case of corresponding high signal (indicating true restricted diffusion) on DWI.

#### Susceptibility effects

**Fig. 9:**
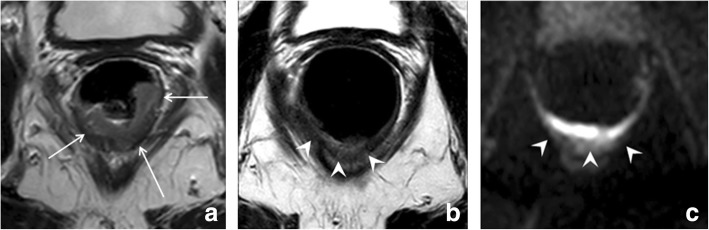
Susceptibility artifacts on DWI. Pre-treatment T2-weighted MRI (**a**), post-CRT T2-weighted (**b**), and post-CRT b1000 diffusion-weighted (**c**) images of a female patient treated for a low rectal tumor (arrows in **a**). On the post-CR images, a large amount of gas is present within the rectal lumen, which causes an artificial pile-up of signal projecting over the dorsal rectal wall at the site of the previous tumor bed (arrowheads in **b** and **c**). If not recognized as an artifact, this signal may be falsely interpreted as tumor

Finally, susceptibility artifacts can be a cause of error on rectal DWI. These artifacts are mainly caused by gas in the rectal lumen and result in distortions and pile up of signal that can hamper correct image interpretation, particularly when projecting over the rectal wall. While large artifacts are generally easily recognized, more subtle artifacts may be mistaken for tumor (example shown in Fig. 9). Current research is investigating how to best prevent these type of artifacts, either by reducing the amount of gas in the rectal lumen (e.g., with endorectal filling or a preparatory micro-enema [7]), or by compensating artifacts through use of alternative methods of DWI image acquisition [43, 44].

### Patterns of response on DWI

**Fig. 10:**
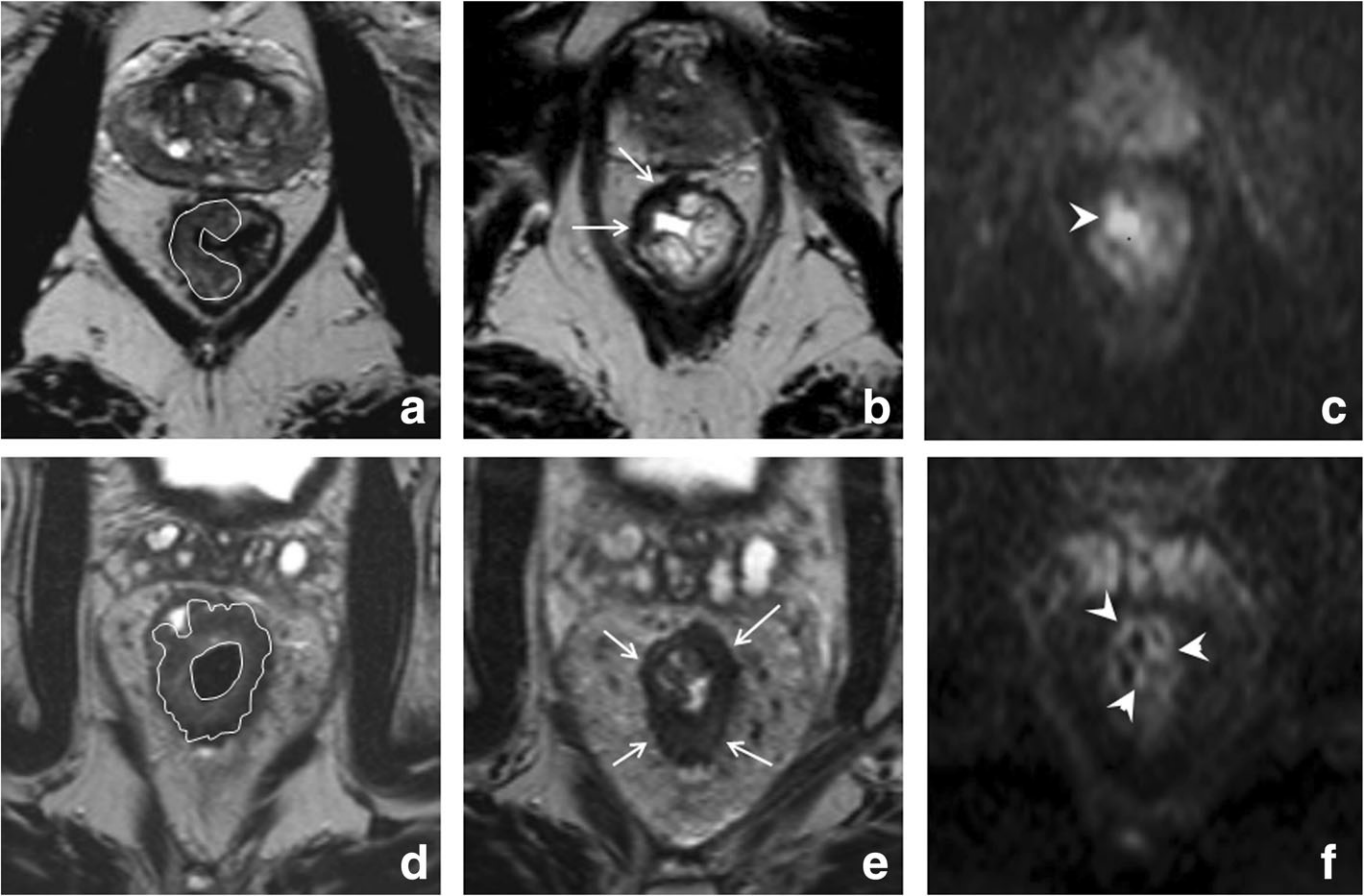
Patterns of response on DWI. Pre-CRT T2-weighted (**a**, **d**), post-CRT T2-weighted (**b**, **e**), and corresponding post-CRT b1000 DWI (**c**, **f**) images of two male patients treated for low rectal tumors. In the upper patient, a semicircular solid mass is visualized before treatment (**a**). After CRT, a focal fibrotic wall thickening is observed (arrows in **b**), without a clear residual mass. On DWI, a focal high signal is observed at the inner margin of the fibrosis (arrowhead in **c**), which was later histopathologically confirmed to be a ypT2 tumor remnant. The patient in the bottom row has a large, irregular circular tumor mass pre-CRT (**d**). After CRT, an irregular and slightly heterogeneous fibrotic wall thickening was observed (arrows in **e**) with small spots of high DWI signal (arrowheads in **f**) scattered throughout the fibrosis. This patient was confirmed to have a ypT3 tumor remnant at histopathology

To prevent misinterpretation of high signal caused by reasons other than residual tumor (see pitfalls described above), it may be helpful to take certain patterns into account when reviewing rectal DWI (Fig. 10). A recent study combined morphologic patterns of fibrosis on T2W-MRI with distinct distribution patterns of high DWI signal within the fibrosis [24]. As described above, primarily semicircular or polypoid tumors tend to regress into a focal fibrotic wall thickening after CRT. In case of residual vital tumor within the fibrosis, the high signal on DWI will typically occur as a focal high signal at the inner margin of the fibrosis (Fig. 10a–c). On the other hand, large circular or irregular tumors tend to show more irregular fibrosis after CRT. In these patients, there is a relative high risk of residual tumor (80%), regardless of the presence of high DWI signal. When present, the DWI signal will typically not present as a single high signal focus but rather more scattered (as small tumor nests) throughout the fibrosis, making it more difficult to detect (Fig. 10d–f). With this pattern approach, a sensitivity of 94% and specificity of 77% was reached to differentiate patients with residual tumor from patients with a complete response, which is higher than that previously reported for studies without a pattern-based evaluation. Alternative approaches combining morphological T2-weighted with DWI patterns have recently been proposed by other authors [45]. Results of these various approaches remain to be compared and validated in prospective settings.

## Lymph node assessment after CRT

It is well-known that nodal staging poses one of the main challenges for radiologists in rectal cancer imaging. Traditionally, radiologists mainly relied on nodal size as the main criterion for malignancy, even though size is known to be an unreliable predictor. Many of the nodal metastases in rectal cancer occur in small-sized nodes while on the other hand false positives frequently occur in reactively enlarged benign nodes. Morphological criteria such as the round shape, irregular border, and heterogeneous signal intensity can help characterize malignant nodes [46], although these criteria can be difficult to assess in small nodes. It is generally acknowledged that performance of MRI for nodal restaging after CRT is better than in the primary staging setting, with negative predictive values of up to 95% to identify ypN0 patients [47]. As a result of CRT, the majority of lymph nodes decrease in size or even completely disappear on MRI. These nodes have a low risk of harboring metastases. Nodes that remain clearly visible after CRT are still at risk [48]. Although the optimal size cut-off after CRT remains a topic of debate, the recent ESGAR guidelines propose a cut-off of 5 mm (short axis) to diagnose yN+ nodes after CRT [2].

**Fig. 11:**
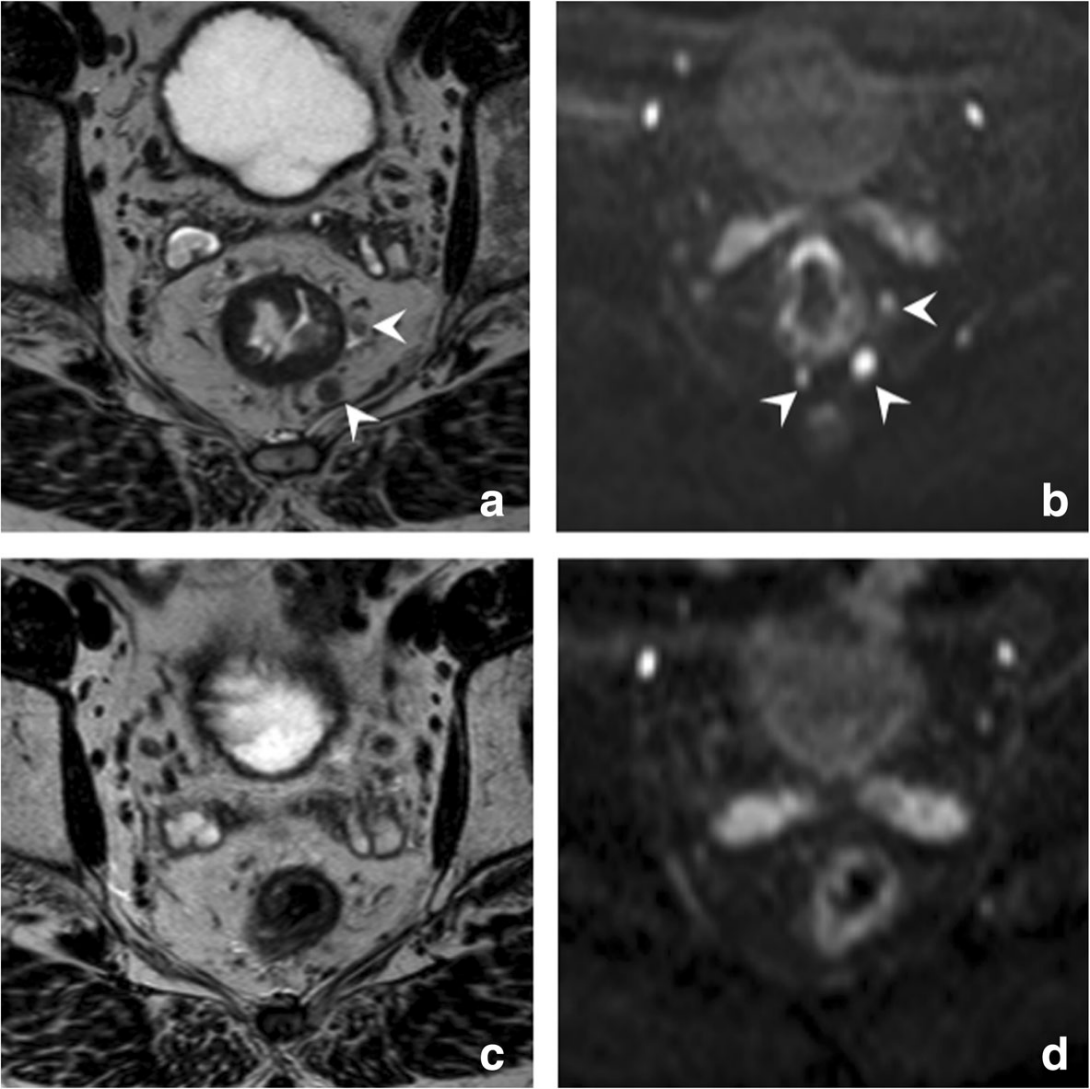
DWI for assessment of lymph nodes. Pre-CRT T2-weighted (**a**), b1000 DWI (**b**), and corresponding post-CRT T2-weighted (**c**) and b1000 DWI (**d**) images of a male patient with a low rectal tumor. On the pre-CRT images, several lymph nodes are visible (arrowheads). Note that the nodes are much easier detectable on DWI and that more nodes can be appreciated on DWI compared to T2-weighted MRI. After CRT, all the lymph nodes have vanished, both on T2W as well as on DWI. The absence of nodes on DWI is a reliable sign indicating a ypN0 stage (which was later confirmed at histopathology after surgery in this patient)

Addition of DWI can be beneficial in the sense that it is a highly sensitive technique to detect the presence of lymph nodes, due to the inherently high cellular density of lymphoid tissue (Fig. 11) [49, 50]. One study reported that the absence of nodes on DWI after CRT can therefore serve as a reliable predictor of a ypN0 status [51]. However, in the majority of patients, one or more nodes remain visible after CRT. In these patients, neither visual nor quantitative assessment of nodal DWI seems to offer significant added benefit compared to standard MRI to differentiate between yN0 and yN+ nodes [52, 53].

## Quantitative functional imaging

In recent years, increasing numbers of reports have focused on quantitative imaging approaches to assess response to CRT in rectal cancer. Although part of this research focusses on use of routine T2-weighted imaging (e.g., T2W tumor volumetry and quantification of T2W signal intensities), most research has focused on use of functional imaging sequences. The most frequently investigated technique is diffusion-weighted MRI.

### Quantitative DWI assessment

**Fig. 12:**
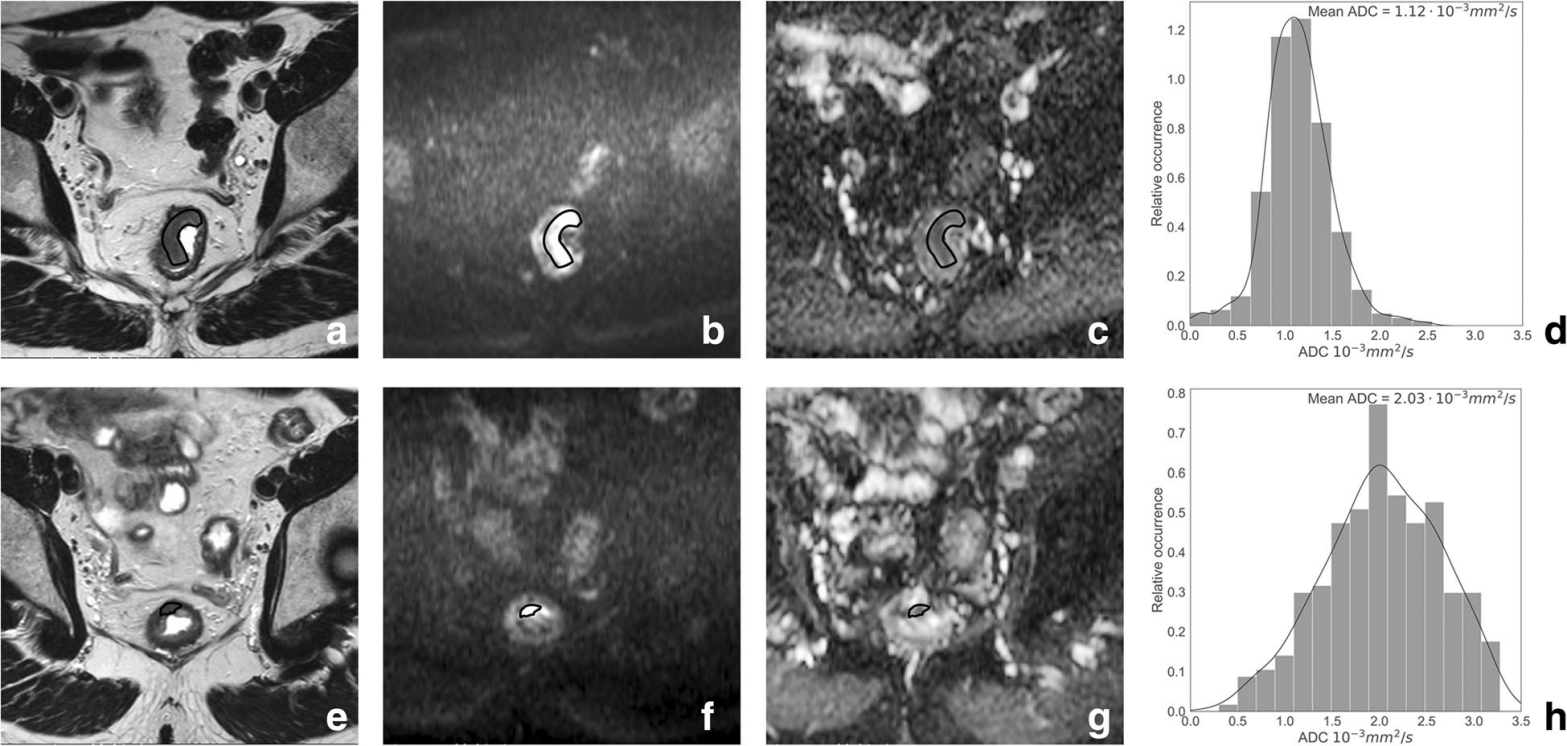
Methods of DWI response evaluation. T2-weighted MRI (**a**, **e**), b1000 DWI (**b**, **f**), ADC map (**c**, **g**), and histograms of ADC values (**d**, **h**) of a patient with a mid-rectal tumor pre- (upper row) and post-CRT (bottom row). The images illustrate different ways DWI can be used to quantitatively assess response: pre-CRT the DWI tumor volume was 11.4 cm^3^ with a corresponding mean ADC of 1.12∙10^−3^ mm^2^/s. Post-CRT the DWI tumor volume decreased to 0.32 cm^3^, while the ADC value increased to 2.03∙10^−3^ mm^2^/s. The histograms show that the distribution of ADC values within the tumor shifted towards more high ADC values after CRT, indicating a good response. At histopathology, a small ypT2 tumor remnant with predominant fibrosis (Mandard tumor regression grade of 2) was found

There are several ways DWI can be used to assess response, in addition to simple visual evaluation (see Fig. 12). The most commonly investigated approach is to quantitatively measure the tumor ADC and determine the change in ADC (ΔADC) as a result of CRT. ADC values typically increase after CRT, reflecting a loss off cell membrane integrity which increases the extracellular space for water diffusion. Both the final ADC post-CRT as well as the ∆ADC have repeatedly been reported to be significantly higher in good-responding patients [54–57], although there is also a minority of studies that found no significant results. In addition, several groups reported higher pre-treatment ADC values to be predictive of a poor response to CRT [58–60], which is believed to be due to the presence of necrosis that makes tumors less susceptible to treatment. Another contributing factor might be the presence of mucinous components within the tumor, since—as described above—mucinous tumors also have a tendency to respond poorly to treatment. Again, other groups found no significant or even conflicting results [54, 56, 57, 61]. Overall, there is quite substantial evidence that ADC has potential value as an imaging biomarker of response, but standardization and large-scale validation are both steps that need to be undertaken before ADC can move forward as a clinical imaging marker. As such, quantification of ADC is currently not recommended in clinical routine [2]. An alternative way to quantitatively use DWI is DWI tumor volumetry. Good results have been reported for the DWI tumor volume (and ∆volume) post-CRT to differentiate between complete responders and patients with residual tumor with reported accuracies of up to 94%, results that significantly outperformed those of conventional T2-weighted tumor volumetry (as well as ADC) in those reports [62–64].

### Other functional imaging and image quantification techniques

Second to DWI, the most commonly researched functional MRI technique is dynamic contrast enhanced (DCE) or ‘perfusion’ MRI. By measuring the inflow of intravenously injected contrast agents into vessels and the leakage of contrast into the extracellular space, DCE-MRI can extract quantitative and semi-quantitative parameters related to tissue perfusion and microvascularity, which have shown significant correlations with response [65–67]. Although already routinely applied in prostate and breast imaging, in rectal cancer, DCE-MRI has so far mainly been applied in research settings and its use is not (yet) advised for clinical routine [2]. Less commonly investigated techniques include MR spectroscopy, magnetization transfer (MT) imaging, and blood oxygenation level-dependent (BOLD) MR. More advanced methods of DWI acquisition such as intravoxel incoherent motion (IVIM) imaging and diffusion kurtosis imaging have also recently been introduced. In addition, there is a growing interest for the use of advanced image post-processing techniques such as Radiomics to extract multiple quantifiable measures (‘features’) from routinely acquired MRI sequences to acquire a radiological tumor phenotype. These methods are to date still in premature stages of research and not yet ready to be adopted in clinics. Since the focus of this pictorial review is on clinical MR methods to assess response, to provide a complete overview of these quantitative approaches would be beyond the scope of the current paper.

## Conclusions

Recent developments in rectal treatment urge the need for an accurate radiological response evaluation. Reduction in volume and fibrotic transformation are the two main signs of response that can be appreciated on morphological (T2-weighted) MRI and used to help guide the treatment strategy after CRT. Morphological MRI is mainly hampered by its inability to discriminate between sterilized fibrosis and fibrosis still containing viable tumor. This limits the performance of MRI to identify complete responders, which is an increasingly important clinical issue given the recent paradigm shift in rectal cancer treatment toward organ-preserving treatments. Addition of diffusion-weighted imaging to the MR protocol improves the performance to discriminate between tumor and fibrosis, but certain pitfalls need to be taken into account. Knowledge on specific patterns of morphology and diffusion signal can help to further optimize diagnostic performance. Image quantification methods are promising and may provide valuable imaging biomarkers to assess response, but to date these methods are still in the research phase and not yet ready to be adopted into clinics.

## References

[1] van de Velde CJ, Boelens PG, Borras JM (2014). EURECCA colorectal: multidisciplinary management: European consensus conference colon & rectum. Eur J Cancer.

[2] Beets-Tan RGH, Lambregts DMJ, Maas M (2018). Magnetic resonance imaging for clinical management of rectal cancer: updated recommendations from the 2016 European Society of Gastrointestinal and Abdominal Radiology (ESGAR) consensus meeting. Eur Radiol.

[3] Maas M, Nelemans PJ, Valentini V (2010). Long-term outcome in patients with a pathological complete response after chemoradiation for rectal cancer: a pooled analysis of individual patient data. Lancet Oncol.

[4] Martens MH, Maas M, Heijnen LA et al (2016) Long-term outcome of an organ preservation program after neoadjuvant treatment for rectal Cancer. J Natl Cancer Inst 108(12). 10.1093/jnci/djw171 10.1093/jnci/djw171 27509881

[5] Habr-Gama A, Gama-Rodrigues J, São Julião GP (2014). Local recurrence after complete clinical response and watch and wait in rectal cancer after neoadjuvant chemoradiation: impact of salvage therapy on local disease control. Int J Radiat Oncol Biol Phys.

[6] van der Valk MJM, Hilling DE, Bastiaannet E et al (2018) Long-term outcomes of clinical complete responders after neoadjuvant treatment for rectal cancer in the International Watch & Wait Database (IWWD): an international multicentre registry study. Lancet 391:2537–2545 10.1016/S0140-6736(18)31078-X 29976470

[7] van Griethuysen JJM, Bus EM, Hauptmann M (2018). Gas-induced susceptibility artefacts on diffusion-weighted MRI of the rectum at 1.5T—effect of applying a micro-enema to improve image quality. Eur J Radiol.

[8] Slater A, Halligan S, Taylor SA, Marshall M (2006). Distance between the rectal wall and mesorectal fascia measured by MRI: effect of rectal distension and implications for preoperative prediction of a tumour-free circumferential resection margin. Clin Radiol.

[9] Sloothaak DA, Geijsen DE, van Leersum NJ (2013). Optimal time interval between neoadjuvant chemoradiotherapy and surgery for rectal cancer. Br J Surg.

[10] West MA, Dimitrov BD, Moyses HE (2016). Timing of surgery following neoadjuvant chemoradiotherapy in locally advanced rectal cancer—a comparison of magnetic resonance imaging at two time points and histopathological responses. Eur J Surg Oncol.

[11] Barbaro B, Vitale R, Leccisotti L (2010). Restaging locally advanced rectal cancer with MR imaging after chemoradiation therapy. Radiographics.

[12] O’Neil MA, Damjanov I (2009). Histopathology of colorectal cancer after neoadjuvant chemoradiation therapy. Open Pathol J.

[13] Martens MH, van Heeswijk MM, van den Broek JJ (2015). Prospective, multicenter validation study of magnetic resonance volumetry for response assessment after preoperative chemoradiation in rectal cancer: can the results in the literature be reproduced?. Int J Radiat Oncol Biol Phys.

[14] Mandard AM, Dalibard F, Mandard JC (1994). Pathologic assessment of tumor regression after preoperative chemoradiotherapy of esophageal carcinoma. Clinicopathologic correlations. Cancer.

[15] Sidiqui MR, Gormly KL, Bhoday J (2016). Interobserver agreement of radiologists assessing the response of rectal cancers to preoperative chemoradiation using the MRI tumour regression grading (mrTRG). Clin Radiol.

[16] Sclafani F, Brown G, Cunningham D (2017). Comparison between MRI and pathology in the assessment of tumour regression grade in rectal cancer. Br J Cancer.

[17] Patel UB, Brown G, Rutten H (2012). Comparison of magnetic resonance imaging and histopathological response to chemoradiotherapy in locally advanced rectal cancer. Ann Surg Oncol.

[18] Patel UB, Taylor F, Blomqvist L (2011). Magnetic resonance imaging-detected tumor response for locally advanced rectal cancer predicts survival outcomes: MERCURY experience. J Clin Oncol.

[19] Gollins S, West N, Sebag-Montefiore D (2018). A prospective phase II study of pre-operative chemotherapy then short-course radiotherapy for high risk rectal cancer: COPERNICUS. Br J Cancer.

[20] Siddiqui MR, Bhoday J, Battersby NJ (2016). Defining response to radiotherapy in rectal cancer using magnetic resonance imaging and histopathological scales. World J Gastroeneterol.

[21] Battersby NJ, Dattani M, Rao S (2017). A rectal cancer feasibility study with an embedded phase III trial design assessing magnetic resonance tumour regression grade (mrTRG) as a novel biomarker to stratify management by good and poor response to chemoradiotherapy (TRIGGER): study protocol for a randomised controlled trial. Trials.

[22] Park SH, Lim JS, Lee J et al (2017) Rectal mucinous adenocarcinoma: MR imaging assessment of response to concurrent chemotherapy and radiation therapy—a hypothesis generating study. Radiology 285:124–133 10.1148/radiol.2017162657 28520513

[23] Lee MA, Cho SH, Seo AN (2017). Modified 3-point MRI-based tumor regression grade incorporating DWI for locally advanced rectal cancer. AJR Am J Roentgenol.

[24] Lambregts D, Delli Pizzi A, Lahaye M (2018). A pattern-based approach combining tumor morphology on MRI with distinct signal patterns on diffusion-weighted imaging to assess response of rectal tumors after chemoradiotherapy. Dis Colon Rectum.

[25] Lambregts DM, Maas M, Bakers FC (2011). Long-term follow-up features on rectal MRI during a wait-and-see approach after a clinical complete response in patients with rectal cancer treated with Chemoradiotherapy. Dis Colon Rectum.

[26] Kim SH, Lee JM, Hong SH (2009). Locally advanced rectal cancer: added value of diffusion-weighted MR imaging in the evaluation of tumor response to neoadjuvant chemo- and radiation therapy. Radiology.

[27] Lambregts DMJ, Vandecaveye V, Barbaro B (2011). Diffusion-weighted MRI for selection of complete responders after chemoradiation for locally advanced rectal cancer: a multicenter study. Ann Surg Oncol.

[28] Foti PV, Privitera G, Piana S (2016). Locally advanced rectal cancer: qualitative and quantitative evaluation of diffusion-weighted MR imaging in the response assessment after neoadjuvant chemo-radiotherapy. Eur J Radiol Open.

[29] Song I, Kim SH, Lee SJ, Choi JY, Kim MJ, Rhim H (2012). Value of diffusion-weighted imaging in the detection of viable tumour after neoadjuvant chemoradiation therapy in patients with locally advanced rectal cancer: comparison with T2 weighted and PET/CT imaging. Br J Radiol.

[30] Sassen S, de Booij M, Sosef M (2013). Locally advanced rectal cancer: is diffusion weighted MRI helpful for the identification of complete responders (ypT0N0) after neoadjuvant chemoradiation therapy?. Eur Radiol.

[31] van der Paardt MP, Zagers MB, Beets-Tan RG, Stoker J, Bipat S (2013). Patients who undergo preoperative chemoradiotherapy for locally advanced rectal cancer restaged by using diagnostic MR imaging: a systematic review and meta-analysis. Radiology.

[32] Krdzalic J, Maas M, Engelen S (2017). MRI can accurately predict sphincter preservation after chemoradiation. Insights Imaging.

[33] Barbaro B, Fiorucci C, Tebala C (2009). Locally advanced rectal cancer: MR imaging in prediction of response after preoperative chemotherapy and radiation therapy. Radiology.

[34] Beets-Tan RG, Beets GL, Vliegen RF (2001). Accuracy of magnetic resonance imaging in prediction of tumour-free resection margin in rectal cancer surgery. Lancet.

[35] MERCURY Study Group (2006). Diagnostic accuracy of preoperative magnetic resonance imaging in predicting curative resection of rectal cancer: prospective observational study. BMJ.

[36] Vliegen RF, Beets GL, Lammering G (2008). Mesorectal fascia invasion after neoadjuvant chemotherapy and radiation therapy for locally advanced rectal cancer: accuracy of MR imaging for prediction. Radiology.

[37] Chand M, Yu S, Swift RI, Brown G (2014). Mucinous carcinoma of the rectum: a distinct clinicopathological entity. Tech Coloproctol.

[38] Yu SK, Chand M, Tait DM, Brown G (2014). Magnetic resonance imaging defined mucinous rectal carcinoma is an independent imaging biomarker for poor prognosis and poor response to preoperative chemoradiotherapy. Eur J Cancer.

[39] Hugen N, van de Velde CJ, de Wilt JH, Nagtegaal ID (2014). Metastatic pattern in colorectal cancer is strongly influenced by histological subtype. Ann Oncol.

[40] Charles-Edwards EM, deSouza NM (2006). Diffusion-weighted magnetic resonance imaging and its application to cancer. Cancer Imaging.

[41] Rao SX, Zeng MS, Chen CZ (2008). The value of diffusion-weighted imaging in combination with T2-weighted imaging for rectal cancer detection. Eur J Radiol.

[42] Lambregts DM, van Heeswijk MM, Delli Pizzi A (2017). Diffusion-weighted MRI to assess response to chemoradiotherapy in rectal cancer: main interpretation pitfalls and their use for teaching. Eur Radiol.

[43] Korn N, Kurhanewicz J, Banerjee S, Starobinets O, Saritas E, Noworolski S (2015). Reduced-FOV excitation decreases susceptibility artifact in diffusion-weighted MRI with endorectal coil for prostate cancer detection. Magn Reson Imaging.

[44] Kyriazi S, Blackledge M, Collins DJ, Desouza NM (2010). Optimising diffusion-weighted imaging in the abdomen and pelvis: comparison of image quality between monopolar and bipolar single-shot spin-echo echo-planar sequences. Eur Radiol.

[45] Santiago I, Barata MJ, Figueiredo N, Pares O, Matos C (2018). The tram track sign: a new, highly specific and reliable sign for the detection of complete response after neoadjuvant therapy in rectal cancer. Insights Imaging.

[46] Brown G, Richards CJ, Bourne MW (2003). Morphologic predictors of lymph node status in rectal cancer with use of high-spatial-resolution MR imaging with histopathologic comparison. Radiology.

[47] Lahaye MJ, Beets GL, Engelen SM (2009). Locally advanced rectal cancer: MR imaging for restaging after neoadjuvant radiation therapy with concomitant chemotherapy. Part II. What are the criteria to predict involved lymph nodes?. Radiology.

[48] Heijnen LA, Maas M, Beets-Tan RG (2016). Nodal staging in rectal cancer: why is restaging after chemoradiation more accurate than primary nodal staging?. Int J Colorectal Dis.

[49] Heijnen LA, Lambregts DM, Mondal D (2013). Diffusion-weighted MR imaging in primary rectal cancer staging demonstrates but does not characterise lymph nodes. Eur Radiol.

[50] Mir N, Sohaib SA, Collins D, Koh DM (2010). Fusion of high b-value diffusion-weighted and T2-weighted MR images improves identification of lymph nodes in the pelvis. J Med Imaging Radiat Oncol.

[51] van Heeswijk MM, Lambregts DM, Palm WM (2017). DWI for assessment of rectal cancer nodes after chemoradiotherapy: is the absence of nodes at DWI proof of a negative nodal status?. AJR Am J Roentgenol.

[52] Lambregts DM, Maas M, Riedl RG (2011). Value of ADC measurements for nodal staging after chemoradiation in locally advanced rectal cancer-a per lesion validation study. Eur Radiol.

[53] Ryu KH, Kim SH, Yoon JH (2016). Diffusion-weighted imaging for evaluating lymph node eradication after neoadjuvant chemoradiation therapy in locally advanced rectal cancer. Acta Radiol.

[54] Ippolito D, Monguzzi L, Guerra L (2012). Response to neoadjuvant therapy in locally advanced rectal cancer: assessment with diffusion-weighted MR imaging and 18FDG PET/CT. Abdom Imaging.

[55] Monguzzi L, Ippolito D, Bernasconi DP, Trattenero C, Galimberti S, Sironi S (2013). Locally advanced rectal cancer: value of ADC mapping in prediction of tumor response to radiochemotherapy. Eur J Radiol.

[56] Barbaro B, Vitale R, Valentini V (2012). Diffusion-weighted magnetic resonance imaging in monitoring rectal cancer response to neoadjuvant chemoradiotherapy. Int J Radiat Oncol Biol Phys.

[57] Nougaret S, Vargas HA, Lakhman Y (2016). Intravoxel incoherent motion-derived histogram metrics for assessment of response after combined chemotherapy and radiation therapy in rectal cancer: initial experience and comparison between single-section and volumetric analyses. Radiology.

[58] Intven M, Reerink O, Philippens ME (2013). Diffusion-weighted MRI in locally advanced rectal cancer: pathological response prediction after neo-adjuvant radiochemotherapy. Strahlenther Onkol.

[59] Jung SH, Heo SH, Kim JW (2012). Predicting response to neoadjuvant chemoradiation therapy in locally advanced rectal cancer: diffusion-weighted 3 Tesla MR imaging. J Magn Reson Imaging.

[60] Sun YS, Zhang XP, Tang L (2010). Locally advanced rectal carcinoma treated with preoperative chemotherapy and radiation therapy: preliminary analysis of diffusion-weighted MR imaging for early detection of tumor histopathologic downstaging. Radiology.

[61] Kremser C, Judmaier W, Hein P, Griebel J, Lukas P, de Vries A (2003). Preliminary results on the influence of chemoradiation on apparent diffusion coefficients of primary rectal carcinoma measured by magnetic resonance imaging. Strahlenther Onkol.

[62] Curvo-Semedo L, Lambregts DM, Maas M (2011). Rectal cancer: assessment of complete response to preoperative combined radiation therapy with chemotherapy—conventional MR volumetry versus diffusion-weighted MR imaging. Radiology.

[63] Ha HI, Kim AY, Yu CS, Park SH, Ha HK (2013). Locally advanced rectal cancer: diffusion-weighted MR tumour volumetry and the apparent diffusion coefficient for evaluating complete remission after preoperative chemoradiation therapy. Eur Radiol.

[64] Lambregts DM, Rao SX, Sassen S (2015). MRI and diffusion-weighted MRI volumetry for identification of complete tumor responders after preoperative chemoradiotherapy in patients with rectal cancer: a bi-institutional validation study. Ann Surg.

[65] Intven M, Monninkhof EM, Reerink O, Philippens ME (2015). Combined T2w volumetry, DW-MRI and DCE-MRI for response assessment after neo-adjuvant chemoradiation in locally advanced rectal cancer. Acta Oncol.

[66] Petrillo M, Fusco R, Catalano O et al (2015) MRI for assessing response to neoadjuvant therapy in locally advanced rectal cancer using DCE-MR and DW-MR data sets: a preliminary report. Biomed Res Int. 2015;2015:514740. 10.1155/2015/514740 PMC4564611 26413528

[67] Martens MH, Subhani S, Heijnen LA (2015). Can perfusion MRI predict response to preoperative treatment in rectal cancer?. Radiother Oncol.

